# Clinical characteristics and mortality predictors among very old patients with pulmonary thromboembolism: a multicenter study report

**DOI:** 10.1186/s12890-023-02824-7

**Published:** 2024-01-10

**Authors:** Xia Zhou, Yuanhua Yang, Zhenguo Zhai, Dingyi Wang, Jieping Lei, Xiaomao Xu, Yingqun Ji, Qun Yi, Hong Chen, Xiaoyun Hu, Zhihong Liu, Yimin Mao, Jie Zhang, Juhong Shi, Zhu Zhang, Sinan Wu, Qian Gao, Xincao Tao, Wanmu Xie, Jun Wan, Yunxia Zhang, Shuai Zhang, Kaiyuan Zhen, Zhonghe Zhang, Baomin Fang, Chen Wang, on behalf of the China pUlmonary thromboembolism REgistry Study (CURES) investigators, Jifeng Li, He Yang, Lan Wang, Haixia Zhou, Maoyun Wang, Xiaohui Wang, Qin Luo, Junping Fan, Jun An, Mian Zeng, Xia Li, Ling Zhu, Yi Liu, Kejing Ying, Guofeng Ma, Chao Yan, Lixia Dong, Wei Zhou, Chong Bai, Wei Zhang, Liangxing Wang, Yupeng Xie, Xiaoying Huang, Chen Qiu, Yazhen Li, Yingyun Fu, Shengguo Liu, Shengqing Li, Jian Zhang, Xinpeng Han, Qixia Xu, Xiaoqing Li, Yingying Pang, Beilei Gong, Ping Huang, Yanwei Chen, Jiming Chen, Guochao Shi, Yongjie Ding, Zhaozhong Cheng, Li Tong, Zhuang Ma, Lei Liu, Luning Jiang, Zhijun Liang, Chaosheng Deng, Minxia Yang, Dawen Wu, Shudong Zhang, Lijun Kang, Fangfei Yu, Xuewei Chen, Dan Han, Shasha Shen, Guohua Sun, Yutao Hou, Baoliang Liu, Xiaohong Fan, Ping Zhang, Ruhong Xu, Zaiyi Wang, Cunzi Yan, Chunxiao Yu, Zhenfang Lu, Jing Hua, Zhenyang Xu, Hongxia Zhang, Jinxiang Wang, Xiaohong Yang, Ying Chen, Yongjun Tang, Wei Yang, Nuofu Zhang, Linli Duan, Simin Qing, Chunli Liu, Lian Jiang, Hongda Zhao, Chengying Liu, Yadong Yuan, Xiaowei Gong, Xinhong Zhang, Chunyang Zhang, Shuyue Xia, Hui Jia, Yunxia Liu, Dongmei Zhang, Yuntian Ma, Lu Guo, Jing Zhang, Lina Han, Xiaomin Bai, Guoru Yang, Guohua Yu, Ruian Yang, Jingyuan Fan, Aizhen Zhang, Rui Jiang, Xueshuang Li, Yuzhi Wu, Jun Han, Jingping Yang, Xiyuan Xu, Baoying Bu, Chaobo Cui, Ning Wang, Yonghai Zhang, Jie Duo, Yajun Tuo, Yipeng Ding, Heping Xu, Dingwei Sun, Xiangyan Zhang, Weijia Liu, Hongyang Wang, Yuan Wang, Aishuang Fu, Songping Huang, Qinghua Xu, Wenshu Chai, Jing Li, Yanping Ye, Wei Hu, Jin Chen, Bo Liu, Lijun Suo, Changcheng Guo, Ping Wang, Jinming Liu, Qinhua Zhao, Le Kang, Jianying Xu, Lifen Zhao, Mengyu Cheng, Wei Duan, Qi Wu, Li Li, Xiuqing He, Yueyue Li, Gang Chen, Yunxia Zhao, Zixiao Liu, Guoguang Xia, Tianshui Li, Nan Chen, Xiaoyang Liu, Tao Bian, Yan Wu, Huiqin Yang, Xiaoli Tang, Yiwen Zhang, Faguang Jin, Yanli Chen, Yanyan Li, Miaochan Lao, Liang Dong, Guangfa Zhu, Wenmei Zhang, Liangan Chen, Zhixin Liang, Liping Cui, Cenfeng Xia, Jin Zhang, Peng Zhang, Lianxiang Guo, Sha Niu, Sichong Yu, Guangjie Liu, Xinmao Wang, Yanhua Lv, Zhenyu Liang, Shaoxi Cai, Shuang Yang, Xinyi Zhang, Jiulong Kuang, Yanyan Ding, Yongxiang Zhang, Xuejun Guo, Yanmin Wang, Jialie Wang, Ruimin Hu, Lin Ma, Yuan Gao, Rui Zheng, Zhihong Shi, Hong Li, Yingqi Zhang, Guanli Su, Zhiqiang Qin, Guirong Chen, Xisheng Chen, Zhiwei Niu, Jinjun Jiang, Shujing Chen, Tiantuo Zhang, Hongtao Li, Jiaxin Zhu, Yuqi Zhou, Yinlou Yang, Jiangtao Cheng, Jie Sun, Yanwen Jiang, Jianhua Liu, Yujun Wang, Ju Yin, Lanqin Chen, Min Yang, Ping Jiang, Hongbo Liu, Guohua Zhen, Kan Zhang, Yixin Wan, Hongyan Tao

**Affiliations:** 1 Shijingshan Teaching Hospital of Capital Medical University, Shijingshan Hospital, Capital Medical University, BeijingBeijing, China; 2 Department of Respiratory and Critical Care Medicine, Beijing Institute of Respiratory Medicine and Beijing Chao-Yang Hospital, Capital Medical University, Beijing, China; 3 National Center for Respiratory Medicine, State Key Laboratory of Respiratory Health and Multimorbidity, National Clinical Research Center for Respiratory Diseases, Institute of Respiratory Medicine, Department of Pulmonary and Critical Care Medicine, Center of Respiratory Medicine, Chinese Academy of Medical Sciences, China-Japan Friendship Hospital, No.2 East Yinghua Road, Beijing, 100029 China; 4 Data and Project Management Unit, Institute of Clinical Medical Sciences, China-Japan Friendship Hospital, Beijing, China; 5 Department of Pulmonary and Critical Care Medicine, Beijing Hospital, Beijing, China; 6 Department of Pulmonary and Critical Care Medicine, The First Affiliated Hospital of Dalian Medical University, Dalian, China; 7 Department of Pulmonary and Critical Care Medicine, West China School of Medicine, West China Hospital, Sichuan University, Chengdu, China; 8 Department of Pulmonary and Critical Care Medicine, The First Affiliated Hospital of Chongqing Medical University, Chongqing, China; 9 Department of Pulmonary and Critical Care Medicine, First Hospital of Shanxi Medical University, Taiyuan, China; 10 Fuwai Hospital, Chinese Academy of Medical Science, National Center for Cardiovascular Diseases, Beijing, China; 11 Department of Pulmonary and Critical Care Medicine, The First Affiliated Hospital of Henan University of Science and Technology, Luoyang, China; 12 Department of Pulmonary and Critical Care Medicine, The Second Hospital of Jilin University, Changchun, China; 13 Department of Pulmonary and Critical Care Medicine, Peking Union Medical College Hospital, Beijing, China; 14 Graduate School of Peking Union Medical College, Chinese Academy of Medical Sciences, Peking Union Medical College, Beijing, China; 15 Peking University China-Japan Friendship School of Clinical Medicine, Beijing, China; 16 Chinese Academy of Medical Sciences and Peking Union Medical College, Peking Union Medical College, Beijing, China; 17 Department of Respiratory Medicine, Capital Medical University, Beijing, China

**Keywords:** Pulmonary thromboembolism, Old, Mortality

## Abstract

**Background:**

Clinical characteristics of patients with pulmonary thromboembolism have been described in previous studies. Although very old patients with pulmonary thromboembolism are a special group based on comorbidities and age, they do not receive special attention.

**Objective:**

This study aims to explore the clinical characteristics and mortality predictors among very old patients with pulmonary thromboembolism in a relatively large population.

**Design and participants:**

The study included a total of 7438 patients from a national, multicenter, registry study, the China pUlmonary thromboembolism REgistry Study (CURES). Consecutive patients with acute pulmonary thromboembolism were enrolled and were divided into three groups. Comparisons were performed between these three groups in terms of clinical characteristics, comorbidities and in-hospital prognosis. Mortality predictors were analyzed in very old patients with pulmonary embolism.

**Key results:**

In 7,438 patients with acute pulmonary thromboembolism, 609 patients aged equal to or greater than 80 years (male 354 (58.1%)). There were 2743 patients aged between 65 and 79 years (male 1313 (48%)) and 4095 patients aged younger than 65 years (male 2272 (55.5%)). Patients with advanced age had significantly more comorbidities and worse condition, however, some predisposing factors were more obvious in younger patients with pulmonary thromboembolism. PaO2 < 60 mmHg, eGFR < 60 mL/min/1.73m2, malignancy, anticoagulation as first therapy were mortality predictors for all-cause death in very old patients with pulmonary thromboembolism. The analysis found that younger patients were more likely to have chest pain, hemoptysis (the difference was statistically significant) and dyspnea triad.

**Conclusion:**

In very old population diagnosed with pulmonary thromboembolism, worse laboratory results, atypical symptoms and physical signs were common. Mortality was very high and comorbid conditions were their features compared to younger patients. PaO2 < 60 mmHg, eGFR < 60 mL/min/1.73m2 and malignancy were positive mortality predictors for all-cause death in very old patients with pulmonary thromboembolism while anticoagulation as first therapy was negative mortality predictors.

**Supplementary Information:**

The online version contains supplementary material available at 10.1186/s12890-023-02824-7.

## Introduction

Venous thromboembolism (VTE) includes deep vein thrombosis (DVT) and pulmonary thromboembolism (PTE) and PTE is the third most common cause of vascular death after myocardial infarction and stroke. In acute phase it can be fatal and it also can lead to chronic condition [1, 2]. However, the outcome may be improved if the disease is timely diagnosed and properly managed. In recent years, considerable reduction in mortality during hospitalization was obtained over the years, which might be attributed to risk stratification-guided management [3].

Though management and diagnostic strategy improve mortality of PTE, it is still health burden for medical and health services. The incidence of PTE increased with age, this is most pronounced among the old [4]. Patients aged 40 years and older are at increased risk compared with younger patients and the risk approximately doubles every decade. Nowadays life expectancy is getting longer, the old population is a large population worldwide, the rate of venous thromboembolism will increase, thereby increasing the health burden [5–7]. The old are not only a large population, but also the main population using medications for chronic disease. Age and comorbidities were already confirmed to be associated with poor outcomes [4]. However, old patients are seldomly recruited in randomized clinical trials due to age, multimorbidity and disabilities [8]. Therefore, evidence-based clinical guidelines do not make recommendations for old people of all ages. Clinicians may treat old patients based on general guidelines and their own experience with uncertainty. There have been several studies focusing on patients with pulmonary embolism over 65 years [9–14]. Data on clinical characteristics, management and outcome of PE in very old patients is limited. Thus, this study aims to explore the clinical characteristics and mortality predictors among very old patients with PTE in a relatively large population.

## Materials and methods

### Patients inclusion

The CURES registry (NCT02943343) involved 100 medical centers across China and this study enrolled consecutive patients aged 18 years and older since 2009. Patients were diagnosed acute PTE with or without DVT through computed tomographic pulmonary angiography, ventilation-perfusion lung scintigraphy, magnetic resonance pulmonary angiography or pulmonary angiography. This study complies with the Declaration of Helsinki and was approved by all participating centers’ ethics committees. All recruited patients sighed written informed consent for their participation in the registry. Diagnostic methods were chosen by physicians of the participating centers, and management decisions were determined at the discretion of the physicians and the actual condition of the patients in accordance with the guidelines.

### Data collection

We collected patients’ data including demographics, risk factors, medical history, symptoms and signs, physical and laboratory examinations, therapeutic management and clinical outcomes of the disease during hospitalization by designated case report forms and then record all data into the electronic data capture system by researchers in each participating centre. Data quality was monitored by local investigators and members from research organization who responsible for quality control.

### Risk stratification and management

Risk stratification for all patients had been calculated by hemodynamic status and sPESI score according to the 2014 ESC/ERS guidelines in our previous study [1, 3]. Primary therapy included anticoagulation, thrombolysis, interventional thrombectomy and surgical embolectomy. Initial anticoagulation therapy referred to when a patient was first given anticoagulant therapy at admission instead of systemic thrombolysis agents, inferior vena cava filter, interventional thrombectomy or surgical embolectomy. We defined condition that when systemic thrombolysis was given prior to any other treatment as initial thrombolysis therapy.

### Grouping, study outcomes and definitions

Patients were divided into three groups: very old (≥ 80 years, as stated elsewhere [15]), old (65–79 years), younger (< 65 years). The primary outcome in this study was the composite of death from any cause during hospitalization. Major bleeding was defined as fatal bleeding, and/or a decrease in hemoglobin levels of greater than 20 g L^−1^ (1.24 mmol L^−1^) or more, or leading to transfusion of two or more units of whole blood or red cells, or intracranial bleeding or other condition according to the International Society on Thrombosis and Haemostasias criteria[16]. The outcome events were assessed by a central adjudication committee.

### Statistical analysis

All analyses were performed using the SPSS (Statistical Product and Service Solutions) version 26.0. Continuous variables were presented as mean ± standard deviation or the median (interquartile range) values, and categorical variables were displayed as number (percentage) values. Variables were compared between groups using independent-samples t test, Chi-squared or Fisher’s exact test depending on the types of the variables. Univariable logistic regression model was performed to assess the association between relevant factors and all-cause mortality. Variables with a significance level of *p* < 0.05 were included in the multivariable logistic regression analysis. Odds ratios and 95% confidence intervals were used to demonstrate the associations.

## Results

Seven thousand four hundred thirty-eight patients diagnosed as acute PTE were included in the CURES registry from January 2009 to December 2015. In those patients enrolled, 609 patients aged equal to or greater than 80 years (male 354 (58.1%)). There were 2734 patients aged between 65 and 79 years (male 1313 (48%)) and 4095 patients aged younger than 65 years (male 2272(55.5%)) (Table 1).

**Table 1:** Demographic characteristic, laboratory results, symptoms, physical examinations

Characteristics	Very old group (*n* = 609)	65-79 years (*n* = 2734)	< 65 years (*n* = 4095)	*P* values
Age(years)	83.9 ± 3.3	72.25 ± 4.18	50.53 ± 11.25	
Female n (%)	354 (58.1%)	1313 (48%)	2272 (55.5%)	< 0.001
BMI	23.1 ± 3.4	23.9 ± 3.6	24.03 ± 3.6	< 0.001
Major comorbidity, n (%)				
Cardiovascular disease n (%)	436 (71.6%)	1716 (62.9%)	1291 (31.6%)	< 0.001
Hypertension n (%)	352 (57.8%)	1372 (50.3%)	946 (23.1%)	< 0.001
Coronary heart disease n (%)	185 (30.4%)	567 (20.8%)	252 (6.2%)	< 0.001
Rheumatic heart disease n (%)	3 (0.5%)	25 (0.9%)	20 (0.5%)	0.087
Cardiomyopathy n (%)	4 (0.7%)	15 (0.5%)	26 (0.6%)	0.896
Heart failure n (%)	65 (10.7%)	191 (7.0%)	106 (2.6%)	< 0.001
Respiratory disease n (%)	265 (43.5%)	876 (32.1%)	888 (21.7%)	< 0.001
Chronic obstructive pulmonary disease n (%)	106 (17.4%)	349 (12.8%)	143 (3.5%)	< 0.001
Pulmonary infection n (%)	114 (18.7%)	346 (12.7%)	400 (9.8%)	< 0.001
Pulmonary Tuberculosis n (%)	44 (7.2%)	99 (3.6%)	83 (2.0%)	< 0.001
Asthma n (%)	11 (1.8%)	36 (1.3%)	49 (1.2%)	0.464
Interstitial lung disease n (%)	25 (4.1%)	69 (2.5%)	36 (0.9%)	< 0.001
Cor pulmonale n (%)	35 (5.7%)	86 (8.2%)	68 (1.7%)	< 0.001
Bronchiectasis n (%)	14 (2.3%)	35 (1.3%)	42 (1.0%)	0.026
Lung cancer n (%)	14 (2.3%)	99 (3.6%)	160 (3.9%)	0.137
Hyperlipidemia n (%)	35 (5.7%)	151 (5.5%)	174 (4.3%)	0.032
Diabetes n (%)	111 (18.3%)	383 (14.1%)	301 (7.4%)	< 0.001
Neurological diseases n (%)	150 (24.8%)	449 (16.5%)	337 (8.3%)	< 0.001
Ischemic stroke n (%)	116 (19.2%)	336 (12.4%)	192 (4.7%)	< 0.001
Hemorrhagic stroke n (%)	20 (3.3%)	48 (1.8%)	71 (1.7%)	0.028
Malignancy n (%)	71 (11.7%)	354 (13%)	476 (11.7%)	0.224
Varicose n (%)	35 (5.7%)	207 (7.6%)	299 (7.3%)	0.278
DVT	221 (37%)	998 (36.9%)	1729 (42.7%)	< 0.001
Liver and kidney disease n (%)	24 (4.0%)	92 (3.4%)	194 (4.8%)	0.021
Liver cirrhosis n (%)	2 (0.3%)	8 (0.3%)	18 (0.4%)	0.624
Chronic hepatitis n (%)	6 (1.0%)	34 (1.2%)	87 (2.1%)	0.009
Chronic nephritis n (%)	11 (1.8%)	26 (1.0%)	33 (0.8%)	0.056
Nephrotic syndrome n (%)	4 (0.7%)	26 (1.0%)	55 (1.3%)	0.171
Diuretics n (%)	2 (0.3%)	23 (0.9%)	40 (1.0%)	0.262
Central venous catheterization	0 (0.0%)	0 (0.0%)	14 (0.4%)	0.005
Smoking n (%)	195 (36.7%)	795 (35.3%)	1269 (37.9%)	0.284
Surgery, trauma, immobilization n (%)	474 (78.0%)	2357 (86.2%)	3567 (87.1%)	< 0.001
**Laboratory examination**				
Platelet count (× 109/L)	195.1 ± 75.2	201.5 ± 77.1	218.9 ± 87.2	< 0.001
Hemoglobin (g/L)	123.3 ± 20.7	126.5 ± 20.0	129.9 ± 22.4	< 0.001
White blood cell > 10*10^9^/L	125 (20.8%)	595 (22.1%)	1215 (30.2%)	< 0.001
PaO2 < 60 mmHg n (%)	114 (21.3%)	578 (24%)	623 (18%)	< 0.001
eGFR < 60 mL/min/1.73m2 n (%)	209 (35.9%)	505 (19.4%)	242 (6.2%)	< 0.001
Elevated BNP or NT-proBNP n (%)	385 (63.2%)	1638 (59.9%)	2296 (56.1%)	< 0.001
Elevated CTN n (%)	357 (58.6%)	1549 (56.7%)	2241 (54.7%)	0.096
**Symptoms and signs**				
Cough n (%)	295 (48.5%)	1172 (43%)	1641 (40.2%)	< 0.001
Sputum n (%)	258 (42.5%)	946 (34.7%)	1189 (29.1%)	< 0.001
Dyspnea n (%)	385 (63.3%)	1862 (68.3%)	2746 (67.2%)	0.062
Chest pain n (%)	158 (26.0%)	895 (32.8%)	1798 (44.0%)	< 0.001
Hemoptysis n (%)	34 (5.6%)	223 (8.2%)	748 (18.3%)	< 0.001
Palpitation n (%)	62 (10.2%)	378 (13.9%)	560 (13.7%)	0.047
Syncope n (%)	50 (8.2%)	274 (10.0%)	453 (11.1%)	0.065
**Physical examinations**				
Temperature > 37.3◦C n (%)	45 (7.4%)	224 (8.2%)	531 (13.0%)	< 0.001
Pulse > 110/min n (%)	42 (7.0%)	227 (8.4%)	469 (11.5%)	< 0.001
SBP < 100 mmHg n (%)	12 (2.0%)	107 (3.9%)	249 (6.1%)	< 0.001
DBP < 60 mmHg n (%)	30 (5.0%)	103 (3.8%)	153 (3.8%)	0.351
Respiratory rate > 20/min n (%)	189 (31.2%)	959 (35.2%)	1530 ( 37.5%)	0.005
Cyanosis n (%)	86 (14.3%)	431 (15.8%)	527 (12.9%)	0.003
Moist crackle n (%)	213 (35.3%)	784 (28.8%)	835 (20.5%)	< 0.001
Wheezing n (%)	85 (14.1%)	242 (8.9%)	184 (4.5%)	< 0.001
Vascular murmur n (%)	3 (0.5%)	6 (0.2%)	19 (0.5%)	0.240
Pleural rub n (%)	1 (0.2%)	15 (0.6%)	29 (0.7%)	0.247
Jugular venous distention n (%)	48 (8.0%)	189 (6.9%)	231 (5.7%)	0.024
Cardiomegaly n (%)	57 (9.5%)	223 (8.2%)	256 (6.3%)	0.001
Tricuspid area murmur n (%)	14 (2.3%)	93 (3.4%)	147 (3.6%)	0.266
P2 increase n (%)	81 (13.4%)	367 (13.5%)	700 (17.2%)	< 0.001
Heart beats under the xiphoid process n (%)	8 (1.3%)	40 (1.5%)	82 (2.0%)	0.172
Abdominal signs n (%)	17 (2.8%)	112 (4.1%)	155 (3.8%)	0.326
Limb sighs n (%)	258 (42.6%)	1030 (37.9%)	1557 (38.4%)	0.093
Leg edema n (%)	243 (40.2%)	913 (33.6%)	1355 (33.4%)	0.004
Arm edema n (%)	5 (0.8%)	31 (1.1%)	44 (1.1%)	808
Leg varicose n (%)	32 (5.3%)	176 (6.5%)	267 (6.6%)	0.482
Homan sigh n (%)	7 (1.2%)	25 (0.9%)	97 (2.4%)	< 0.001
Gastrocnemius tenderness n (%)	5 (0.8%)	58 (2.1%)	187 (4.6%)	< 0.001

Surgery and trauma in 3 months, Immobilization more than 3 days; P2: The second heart sound of the pulmonary valve

*eGFR* Estimated glomerular filtration rate, assessed by CKD-EPI formula, *BMI* Body mass index, *SBP* Systolic blood pressure, *DBP* Diastolic blood pressure, *DVT* Deep vein thrombosis, *PaO*_*2*_ Arterial blood gas oxygen partial pressure, *CTN* Cardiac troponin

Patients with advanced age had significantly more comorbidities (including hypertension, coronary heart disease, heart failure, COPD (chronic obstructive pulmonary disease), pulmonary infection, pulmonary tuberculosis, interstitial lung disease, cor pulmonale, bronchiectasis, hyperlipidemia, diabetes, neurological diseases, liver and kidney disease, *p* < 0.05) and some predisposing factors were more common in younger patients with PTE (surgery and trauma in 3 months, immobilization more than 3 days, central venous catheterization, *p* < 0.05). Younger patients were more often diagnosed as PTE accompanied by DVT.

Patients with advanced age had more worse condition: lower BMI (body mass index), lower platelet count, lower hemoglobin, lower PaO_2_, lower eGFR, higher cardiac biomarkers, *p* < 0.05) (Table 1), old patients more often presented with clinical manifestations like cough and sputum. The analysis found that younger patients were more likely to have chest pain, hemoptysis (the difference was statistically significant) and dyspnea triad. Though not significant, there is a tendency for younger patients to be more prone to syncope when VTE occurs. When a physical examination was performed, temperature > 37.3◦C, pulse > 110/min, SBP (systolic blood pressure) < 100 mmHg, respiratory rate > 20/min, P2 increase, positive Homan sigh and gastrocnemius tenderness were more frequent in younger patients. In old and very old patients, signs included cyanosis, moist crackle, wheezing, cardiomegaly, leg edema and jugular venous distention were more common than that in younger patients.

High risk PTE was more common in younger patients, almost twice as often as that in very old patients. The number of patients who received thrombolysis as the initial treatment was 13 (2.2%) in very old patients, 286 (10.7%) in group aged 65-79 years and 607 (15.1%) in younger patients respectively. The main treatment in all three groups was anticoagulation. All cause death increased from 2.3% to 7.4% with age (*p* < 0.001). There was a trend that the rate of bleeding increased with age but not significant. There was no significant difference in the incidence of major bleeding (Table 2).

**Table 2:** Baseline treatment, outcomes

Items	≥ 80 years (*n* = 609)	65-79 years (*n* = 2734)	< 65 years (*n* = 4095)	*P* values
High risk n (%)	15 ( 2.5%)	110 (4.0%)	185 (4.5%)	0.155
Thrombolytic therapy as initial treatment n (%)	13 (2.2%)	286 (10.7%)	607 (15.1%)	< 0.001
Anticoagulation as initial treatment n (%)	524 (86%)	2304 (84.3%)	3395 (82.9%)	0.083
Surgery thrombectomy n (%)	1 (0.2%)	19 (0.7%)	31 (0.8%)	0.258
Interventional thrombectomy n (%)	0 (0.0%)	13 (0.5%)	13 (0.3%)	0.153
IVC filter n (%)	26 (4.4%)	112 (4.2%)	248% (6.2)	0.001
INR up to the standard when discharge n (%)	276 (73.2%)	1470 (75%)	2282 (76.9%)	0.233
All cause death n (%)	45 (7.4%)	115 (4.2%)	93 (2.3%)	< 0.001
Bleeding n (%)	32 (7.1%)	127 (6.4%)	176 (6.2%)	0.723
Major bleeding n (%)	10 (2.2%)	40 (2.0%)	63 (2.2%)	0.901
Days of stay (day)	15 (11, 21)	14 (10, 20)	15 (10, 20)	0.072
Treatment interval (day)	8 (4,14)	8 (4,14)	7 (3,13)	0.107

*IVC* Inferior vena cava, *INR* International normalized ratio

**Fig. 1:**
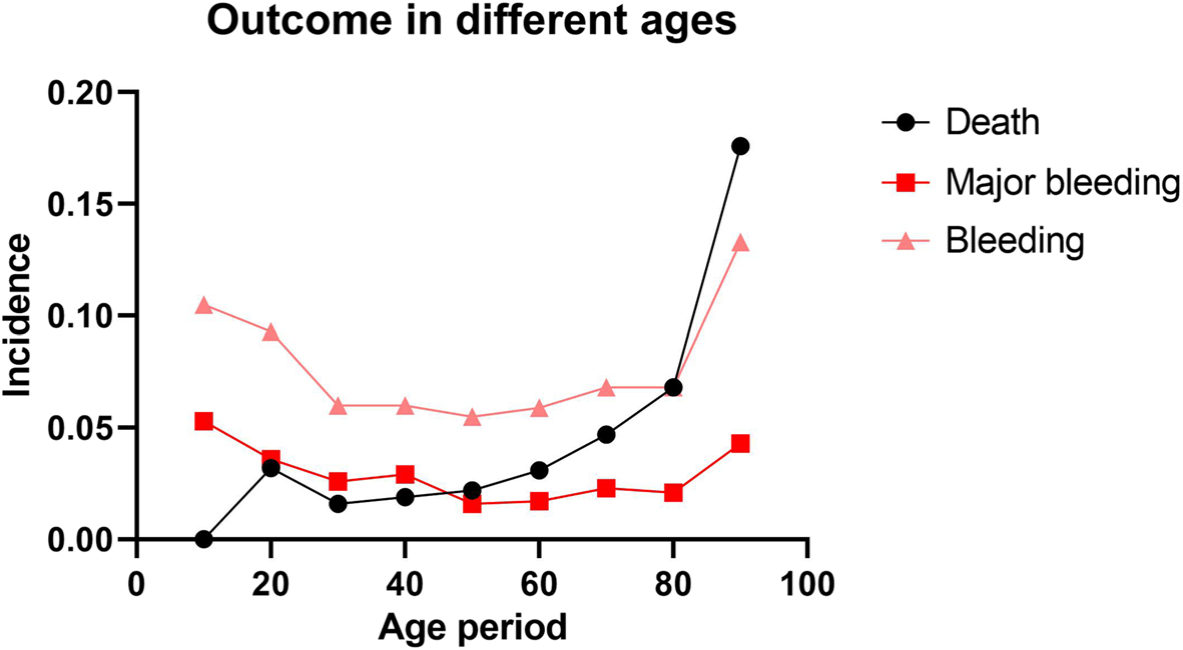
Outcome in different ages. Death *P* < 0.001; Major bleeding *P* = 0.395; Bleeding *P* = 0.473

The outcomes of patients at different age period were shown in the Fig. 1. It could be seen that the mortality rate of patients with PTE increased significantly with the increase of age (*p* < 0.001), while there was no statistical difference in bleeding events and major bleeding events at different age period.

### Comparison between survival and death groups in very old patients

The demographic characteristic showed that very old patients with a prognosis of death had lower BMI (21.6 ± 2.8 vs 23.2 ± 3.4, *p* = 0.002), more comorbid malignancy (7 (15.6%) vs. 36 (6.4%), *p* = 0.021) and anemia (20 (46.5%) vs. 152 (27.8%), *p* = 0.009). They had worse laboratory results: PaO2 < 60 mmHg (17 (47.2%) vs 97 (19.5%), *p* < 0.001), eGFR < 60 mL/min/1.73m2 (23 ( 56.1%) vs 186 ( 34.4%), *p* = 0.005). when physical examination was compared, very old patients with a prognosis of death showed more conditions such as pulse > 110/min (7 ( 15.9%) vs 35 ( 6.3%),*p* = 0.016), SBP < 100 mmHg (3 (6.7%) vs 6 (1.6%), *p* = 0.019), DBP (diastolic blood pressure) < 60 mmHg (6 (13.3%) vs24 (4.3%), *p* = 0.007) (Table 3).

**Table 3:** Demographic characteristic, laboratory results, symptoms, physical examinations

Characteristics	Survival (*n* = 654)	Death (*n* = 45)	*P* values
**Age (years)**	83.9 ± 3.3	85.6 ± 3.8	0.001
**Female n (%)**	329 (58.3%)	25 (55.6%)	0.716
**BMI**	23.2 ± 3.4	21.6 ± 2.8	0.002
**Major comorbidity, n (%)**			
Cardiovascular disease n (%)	400 (70.9%)	36 (80%)	0.194
Respiratory disease n (%)	243 (43.1%)	22 (48.9%)	0.450
Neurological diseases n (%)	137 (24.5%)	13 (28.9%)	0.508
Malignancy n (%)	36 (6.4%)	7 (15.6%)	0.021
Varicose n (%)	34 (6.0%)	1 (2.2%)	0.291
Liver and kidney disease n (%)	20 (3.6%)	4 (8.9%)	0.077
Surgery in 3 months n (%)	42 (7.4%)	4 (8.9%)	0.725
Trauma in 3 months n (%)	49 ( 8.7%)	4 (8.9%)	0.966
Central venous catheterization	0 (0.0%)	0 (0.0%)	-
Smoking n (%)	182 (37.1%)	13 (31.7%)	0.494
Immobilization more than 3 days n (%)	400 (73.9%)	27 (62.8%)	0.113
**Laboratory examination**			
Anemia	152 (27.8%)	20 (46.5%)	0.009
Thrombocytopenia	37 (6.7%)	6 (13.6%)	0.086
White blood cell > 10*10^9^/L	112 (20.1%)	13 (30.2%)	0.115
PaO_2_ < 60 mmHg n (%)	97 (19.5%)	17 (47.2%)	< 0.001
eGFR < 60 mL/min/1.73m2 n (%)	186 (34.4%)	23 (56.1%)	0.005
Elevated cardiac biomarkers n (%)	266 (47.2%)	25 (55.6)	0.278
Elevated BNP or NT-proBNP n (%)	35 (63.1%)	29 (64.4%)	0.859
Elevated CTN n (%)	327 (58%)	30 (66.7%)	0.255
**Symptoms and signs**			
Cough n (%)	273 (48.5%)	22 (48.9%)	0.959
Sputum n (%)	237 (42.1%)	21 (46.7%)	0.551
Dyspnea n (%)	357 (63.4%)	28 (62.2%)	0.874
Chest pain n (%)	145 (25.8%)	13 (28.9%)	0.645
Hemoptysis n (%)	30 (5.3%)	4 (8.9%)	0.317
Palpitation n (%)	57 (10.1%)	5 (11.1%)	0.833
Syncope n (%)	47 (8.3%)	3 (6.7%)	0.693
Temperature > 37.3◦C n (%)	42 (7.5%)	3 (6.7%)	0.842
Pulse > 110/min n (%)	35 (6.3%)	7 (15.9%)	0.016
SBP < 100 mmHg n (%)	6 (1.6%)	3 (6.7%)	0.019
DBP < 60 mmHg n (%)	24 (4.3%)	6 (13.3%)	0.007
Respiratory rate > 20/min n (%)	174 (31.0%)	15 (34.1%)	0.672

*eGFR* Estimated glomerular filtration rate, *BMI* Body mass index, *SBP* Systolic blood pressure, *DBP* Diastolic blood pressure, *DVT* Deep vein thrombosis, *PO*_*2*_ Blood gas oxygen partial pressure, *CTN* Cardiac troponin

There were more high risk PTE in very old patients with outcome of death, almost 11 times as often as that in survival patients. The main treatment in two groups was anticoagulation. Major bleeding occurred more often in very old patients with outcome of death (11.5% vs. 1.6%) (Table 4).

**Table 4:** Risk stratification, baseline treatment, outcomes, bleeding

Items	Survival (*n* = 564)	Death (*n* = 45)	*P* values
High risk n (%)	8 (53.3%)	7 (46.7%)	< 0.001
Intermediate-high risk n (%)	249 (92.2%)	21 (7.8%)	< 0.001
Intermediate-low risk n (%)	307 (94.8%)	17 (5.2%)	< 0.001
Initial thrombolytic therapy n (%)	6 (1.1%)	2 (4.4%)	0.055
Initial anticoagulation n (%)	495 (87.8%)	29 (64.4%)	< 0.001
Surgery thrombectomy n (%)	1 (0.2%)	0 (0.0%)	0.775
Interventional thrombectomy n (%)	0 (0.0%)	0 (0.0%)	-
IVC filter n (%)	25 (4.6%)	1 (2.3%)	0.467
Bleeding n (%)	28 (6.6%)	4 (15.4%)	0.092
Major bleeding n (%)	7 (1.6%)	3 (11.5%)	0.001
≥ 3 comorbidities n (%)	134 (23.8%)	19 (42.2%)	0.006

In logistic-regression analysis, multiple-comorbidity in very old patients was not an influencing factor for death. Univariate logistic-regression analysis found chronic nephritis, age, malignancy, anemia, PaO2 < 60 mmHg, eGFR < 60 mL/min/1.73m^2^, pulse ≥ 110 bpm, SBP < 100 mmHg and DBP < 60 mmHg might be influencing factors of death in very old patients. Thus, they were analyzed in multivariate logistic-regression analysis. The results showed that PaO2 < 60 mmHg ( OR 0.216, 95% CI 0.094–0.497, *p* < 0.001), eGFR < 60 mL/min/1.73m^2^ (OR 0.361, 95%CI 0.160–0.814, *p* = 0.014), malignancy (OR 0. 245, 95%CI 0.091–0.658, *p* = 0.005), anticoagulation as first therapy (OR 3.826, 95%CI 1.511–9.688, *p* = 0.005) were mortality predictors for all-cause death in very old patients with pulmonary embolism (Table 5).

**Table 5:** Univariate and multivariate logistic-regression analysis for influencing factors of death in very old

Variables	Univariate Logistic-regression Analysis				Multivariate Logistic-regression Analysis			
	**P**	**OR**	**95% C.I**		**P**	**OR**	**95% C.I**	
			**Lower**	**Upper**			**Lower**	**Upper**
Age	0.024	0.34	0.133	0.869	0.470	0.576	0.129	2.567
BMI < 18	0.138	0.498	0.198	1.251				
Malignancy, n (%)	0.000	3.537	1.757	7.120	0.005	0.245	0.091	0.658
Liver and kidney disease, n (%)	0.060	2.949	0.953	9.118	0.317	0.463	0.103	2.089
Anamia, n (%)	0.011	2.260	1.206	4.233	0.080	0.483	0.214	1.092
Platelet < 100 × 10^9^/L, n (%)	0.094	2.202	0.875	5.544				
PaO2 < 60 mmHg, n (%)	0.000	3.699	1.854	7.381	0.000	0.216	0.094	0.497
eGFR < 60 mL/min/1.73m2, n (%)	0.006	2.439	1.284	4.633	0.014	0.361	0.160	0.814
Pulse ≥ 110Bpm, n (%)	0.020	2.827	1.176	6.797	0.393	0.580	0.167	2.021
SBP < 100 mmHg, n (%)	0.031	4.389	1.145	16.825	0.931	0.912	0.115	7.243
DBP < 60 mmHg, n (%)	0.011	3.442	1.329	8.917	0.094	0.281	0.063	1.242
Thrombolysis as initial therapy, n (%)	0.078	4.326	0.847	22.079				
Anticoagulation as initial therapy, n (%)	0.000	0.253	0.131	0.489	0.005	3.826	1.511	9.688
≥ 3 comorbidities n (%)	0.007	2.345	1.258	4.370	0.179	0.547	0.227	1.318

## Discussion

Our study results showed that in those 7,438 patients, 609 patients aged equal to or greater than 80 years (58.1%). As a result of population aging, the number of older patients is increasing, especially patients with PTE which its incidence increases with age. Recommendations for older patients on common cardiovascular diseases in current guidelines of the European Society of Cardiology are missing or scarce, let alone PTE [2, 15]. Guidelines rarely provide advice to older patients mainly because older patients were seldomly recruited in previous clinical trials due to age, multimorbidity and disabilities and thus limited evidence concerning diagnosis and treatment of those older patients was summarized [8].

In this study, older patients had significantly more comorbidities but some predisposing factors for PTE such as surgery, trauma, immobilization, central venous catheterization were more common in younger patients. In addition, PTE accompanied by deep vein thrombosis (DVT) were more often diagnosed in younger patients. Such results remind us that older patients with PTE are more likely to develop “unprovoked” PTE, which was in accordance with previous study (old ≥ 65 years) [9]. Older patients may be less prone to PTE due to deep venous embolism than younger patients. Besides, we also found that old patients had symptoms like cough and sputum more frequent than that in their younger counterpart patients. In contrast, chest pain, hemoptysis and dyspnea triad were less common in very old patients. Another research found patients with PTE aged 80 and older were more likely to show syncope (10% vs 6%) at presentation than those younger than 80 [17]. This number was reversed in our study but it was not statistically significant. In our results, syncope was more frequent in younger (8.2% in very old vs.10.0% in 65–79 years vs.11.1% in younger patients), this was consistent with their high-risk distribution (2.5% high risk in very old vs.4.0% high risk in 65–79 years vs.4.5% high risk in younger patients). Thus, we can conclude that the old patients are less likely to show the typical clinical manifestations of PTE that we have considered in the past, but may often present with symptoms like aggravation of underlying disease, for example, COPD and cardiac insufficiency.

In previous study, population aging and high comorbidity were risk factors for PTE and old patients presented with syncope and dyspnea more frequently than in younger patients [11]. In our age stratified study, though not significant, there is a tendency for younger patients to have syncope and dyspnea when PTE occurs. Very old patients have high comorbidity and less typical symptoms. In recent years, many studies have been conducted to help diagnose PTE properly and have improved guidelines. About the old patients, some scholars have suggested that the application of both a fixed higher D-dimer cutoff (1000 ng/mL) and the age-adjusted threshold would increase the specificity of D-dimer assay for excluding PTE and do not reduce sensitivity [18]. When a physical examination was performed, changes in vital signs were more frequent in younger patients and the very old often present with clinical manifestations that seemed to be related to underlying diseases such as moist crackle, wheezing and so on. Nonspecific manifestations and laboratory results might be erroneously attributed to common diseases or to age itself, thus can delay the diagnosis and even misguide treatment [19]. Nonspecific manifestations and laboratory results widened the spectrum of differential diagnosis of PTE in the older, and high clinical suspicion is needed to prevent delays in diagnosis [20]. All these suggest that it is precisely at this stage of suspicion during diagnosis and management of PTE that older people and younger people have different questions to consider. Given the convenience of the current inspection, someone may add check items to reduce missed diagnosis, but this strategy obviously can’t be promoted among all old patients not only because of the cost, but also because the potential renal toxicity of intravenous contrast and the high incidence of renal dysfunction in the old patients themselves [21–23]. Thus, finding a balance between under-suspicion and over-suspicion of PTE is a particularly challenging issue for those very old patients with higher risk of contrast nephropathy.

In our study, age stratified prognosis showed that mortality increased every ten years. All cause death increased from 2.3% to 7.4% with age group (*p* < 0.001). Beside the impact of age itself on mortality, high comorbidity rate such as changes in cardiac function and renal function due to various diseases and age-related fragility all contribute to poor prognosis of old patients [10, 24]. In clinical practice, in fear of bleeding events, some physicians may mistake advanced age and comorbidity as a contraindication to treatment like anticoagulation and thrombolysis [25]. 15.1% of the younger patients were given thrombolysis as the initial treatment but only 2.2% of the very old patients had thrombolysis as the initial treatment. These practice lead to the higher morbidity and mortality associated with PTE in the old than in younger patients [19]. Indeed, results in our research showed no significant difference in major bleeding incidence between age stratified groups, there was a trend that the rate of bleeding increased with age groups but not significant, which was not consistent with previous study [9]. One reason is that the management of pulmonary embolism has improved in recent years [3, 26]. The participating centers in this study choose appropriate treatment strategy and carry out strict control of indications and contraindications. In addition, anticoagulation was administrated according to age and renal function. In some cases, we aimed for an INR level of 1.8–2.5 in old patients instead of an INR level of 2.0–3.0 to avoid bleeding. In RIETE study, the 3.7% incidence of fatal PE in patients aged ≥ 80 years old outweighed the 0.8% of fatal bleeding [27]. In this study, appropriate therapy also did not increase the incidence of bleeding in very old patients. In earlier study [17], Moutzouris JP et.al published that no difference in short-term mortality was found between octogenarians and their younger counterpart. With appropriate assessment of the condition and appropriate choice of treatment to reduce the risk of bleeding, there seems to be more reason to concern about severity of pulmonary embolism itself in old patients rather than to treat advanced age as a contraindication for fear of bleeding.

In this study, very old patients with a prognosis of death had lower BMI, more malignancy and anemia, worse laboratory results and higher incidence of high-risk PTE. Besides, PAO2 < 60 mmHg, eGFR < 60 mL/min/1.73m^2^, malignancy and whether anticoagulation as first therapy were mortality predictors for all-cause death in very old patients with PTE. The choice of anticoagulation as first treatment strategy is beneficial to the prognosis of very old patients during hospitalization. In another retrospective cohort study, researchers found that, in emergency department, mortality is high in an old population with a clinically suspected PTE. They suggest that sPESI scoring, even combined with cardiac troponin testing, is not sufficient to predict mortality in old patients [28]. Perhaps more and better larger studies are needed to promote the development of better risk stratification tools for old and very old patients.

### Limitations

The main limitation of our study is that we enrolled consecutive patients with confirmed diagnosis of PTE. Those patients who died rapidly in the emergency department with a high suspicion of pulmonary embolism death were not included. Therefore, difference of clinical manifestation and outcome of PTE in real world between younger, old and very old patients was not concluded. Since some results were not consistent with previous study, our results need to be confirmed in larger cohorts. The reason only 2 individuals (4.4%) in the death group received thrombolysis despite 7 individuals (15.6%) being classified as high risk was not recorded in our date. In high-risk patients, patients themselves or relatives may refuse thrombolysis because of fear for bleeding. If the physician assesses the very old patient at a very high risk of bleeding, they may not carry out thrombolysis. Our database failed to record the specific causes. Another limitation is that, in multicenter study, some specific causes of death were not retrievable and no definite conclusions can be drawn on relationship between treatment and mortality.

## Conclusion

We conclude that in very old population diagnosed with PTE, worse laboratory results, atypical symptoms and physical sighs were common. Mortality was very high and comorbid conditions were their features compared to younger patients. PaO2 < 60 mmHg, eGFR < 60 mL/min/1.73m2 and malignancy were positive mortality predictors for all-cause death in very old patients with PTE while anticoagulation as first therapy was negative mortality predictors. After reasonable choice of treatment, the incidence of bleeding did not significantly increase with age. The very old patients should receive more attention than the young patients because of their comorbidities and frailty, rather than being excluded from clinical research that influences guidelines, and should not be considered as a contraindication for examination and treatment.

### Supplementary Information

**Additional file 1: Supplement.** Echocardiography in very old patients with PTE.

## Data Availability

The original contributions presented in the study are included in the article, further inquiries can be directed to the corresponding authors.

## Abbreviation

CURES: The China pUlmonary thromboembolism REgistry Study
PTE: Pulmonary thromboembolism
VTE: Venous thromboembolism
DVT: Deep vein thrombosis
sPESI: Simplified pulmonary embolism severity index
COPD: Chronic obstructive pulmonary disease
IVC: Inferior vena cava
INR: International normalized ratio
PaO2: Arterial blood gas oxygen partial pressure
eGFR: Estimated glomerular filtration rate
BMI: Body mass index
SBP: Systolic blood pressure
DBP: Diastolic blood pressure
CTN: Cardiac troponin
