# Supplementary material for: Clinical characteristics and mortality predictors among very old patients with pulmonary thromboembolism: a multicenter study report
Source: BMC Pulm Med. 2024 Jan 10;24:26. doi: 10.1186/s12890-023-02824-7 (PMC10782748; doi:10.1186/s12890-023-02824-7)
Supplement: Supplementary file 1 — Additional file 1: Supplement. Echocardiography in very old patients with PTE. [file 12890_2023_2824_MOESM1_ESM.docx]

| **Items** | **Survival** | | **Death** | **P values** |
| --- | --- | --- | --- | --- |
| Right atrial internal dimension | | 40.375±7.990 | 36.800±6.169 | 0.194 |
| Left atrial internal dimension | | 37.653±7.0614 | 36.000±6.284 | 0.023 |
| Right ventricular internal dimension | | 30.668±8.012 | 25.825±5.409 | 0.238 |
| Left ventricular internal diameter | | 43.370±9.168 | 38.667±5.131 | 0.388 |
| Systolic pulmonary arterial pressure | | 53.029±19.132 | 60.160±16.187 | 0.251 |

Supplement：Echocardiography in very old patients with PTE.
